# Fire on the Hearth

**Published:** 1876-10

**Authors:** 


### "*FIRE ON THE HEARTH.*"

We have a new stove in our office, 137 Eighth Street, and thus far we are so well pleased with its operations that we feel desirous of making its merits known. To be sure, we have not submitted it to any severe tests as yet, for our autumn days do not demand extensive or continuous fires; but we have used it sufficiently to discover that it performs one important function which is claimed for it by its inventor—*it ventilates while it warms.*

The Open Stove Ventilating Company, at 107 Fulton Street, of whom we bought the stove, claim for this heating device that it will warm the air at remote points in the apartment as perfectly as that in near proximity to the stove. They line their store with thermometers, and only a trifling variation is discovered in any of them.

These stoves are quite ornamental in appearance, and are of four sizes—the largest being sufficient to warm a room 20 by 50 feet, and 12 feet high. The arrangement is such that a tight stove or an open fireplace is secured at will. Provision is made for a supply of pure air from without, in localities occupied by many people. On the whole we are pleased with our new stove, and if it works as well as it now promises, we will have something more to say about it, for the benefit of our readers. If it works badly, we will surely speak of that fact, also.

